# The Optimization of Process Parameters and Characterization of High-Performance CF/PEEK Composites Prepared by Flexible CF/PEEK Plain Weave Fabrics

**DOI:** 10.3390/polym11010053

**Published:** 2018-12-31

**Authors:** Chunrui Lu, Nuo Xu, Ting Zheng, Xin Zhang, Hanxiong Lv, Xue Lu, Lin Xiao, Dongxing Zhang

**Affiliations:** School of Materials Science and Engineering, Harbin Institute of Technology, Harbin 150001, China; xunuo_hit@163.com (N.X.); zthappy1127@gmail.com (T.Z.); zhangxin1994_hit@163.com (X.Z.); hitmse.hanson@gmail.com (H.L.); luxue2014hgd@126.com (X.L.)

**Keywords:** flexible CF/PEEK plain weave fabric, processing parameters, mechanical properties

## Abstract

Continuous carbon fiber (CF)-reinforced poly (ether ether ketone) (PEEK) composites have excellent mechanical properties, but their processing techniques are limited. Therefore, we promoted a braiding method based on the hybrid fiber method by hot-compacting CF/PEEK plain weave fabrics to solve the problem of difficult wetting between CF and PEEK. Four parameters—melting temperature, molding pressure, crystallization temperature and the resin contents—were investigated for optimized fabrication. After studying the melting range, thermal stability and the contact angle of PEEK under different temperatures, the melting temperature was set at 370 °C. An ultra-depth-of-field 3D microscope was adopted to investigate the effects of molding pressure in the melting stage. The tensile strength or modulus along and perpendicular to the carbon fiber direction and crystallinity under different crystallization temperatures were analyzed. As a result, the sample crystalized at 300 °C showed an excellent tensile properties and crystallinity. The increased mass ratio of PEEK ranging from 50.45% to 59.07% allowed for much stronger interfacial strength; however, the higher resin content can lead to the dispersion of CFs, loss of resin and the formation of defects during processing. Finally, the optimal resin mass content was 59.07%, with a tensile strength of 738.36 ± 14.49 MPa and a flexural strength of 659.68 ± 57.53 MPa. This paper studied the optimized processing parameters to obtain better properties from CF/PEEK plain weave fabrics and to further broaden the specific applications of CF/PEEK composites, demonstrating a new direction for its fabrication.

## 1. Introduction

With the increasing demand for high-performance composites in aerospace, automobile and other structural applications in engineering, a large amount of effort has been devoted to the study and development for fiber-reinforced thermoplastic composites [1,2,3,4]. Among these, carbon fiber/poly (ether ether ketone) (CF/PEEK) composites have attracted wide attention owing to the superior properties of the semi-crystalline PEEK matrix [5,6,7]. PEEK is a special engineering plastic with excellent physical and chemical properties and processing properties. It has comprehensive properties such as high heat resistance, radiation resistance, self-lubrication, good dimensional stability and excellent electrical properties. The most prominent advantage is its toughness and damage resistance, as well as excellent creep resistance and fatigue resistance. It has good melting fluidity and thermal stability beyond its melting point, and the excellent processing performance allows its fabrication by various methods. Therefore, it has been increasingly applied in the industrial field.

Composite materials with better properties can be obtained by the combination of PEEK and CF. CF can act as a heterogeneous nucleating agent to promote the crystallization of PEEK in composites [8,9,10]. However, due to the high melting viscosity of PEEK itself and its insolubility in many solvents, the preparation of continuous CF/PEEK composites has always been a scientific problem to be solved. At present, CF/PEEK composites are usually prepared by purchasing prepreg from abroad and then hot-pressing it [11]. However, this method has limitations, as CF/PEEK prepreg is a composite fabricated by impregnating continuous fibers with resin matrix under strictly controlled conditions and already possesses certain mechanical properties. When preparing CF/PEEK composites with complex shapes, it is easy to destroy the continuity of the prepreg itself and cause the distribution or discontinuity of reinforced CFs, resulting in a decrease in the mechanical properties. Hence, composites fabricated from CF/PEEK plain weave fabric, which is a flexible premix or pre-prepreg, are promising both in scientific research and practical applications.

Research on fiber hybrid composites began several decades ago. Fiber hybrid composites are structural materials that are formed by a textile matrix and textile reinforcement through weaving, braiding or knitting, etc. ([12,13]). As with the development of polymer fibers, the interest in textile composites is growing due to the requirements of several industries ([14,15]). The NASA Advanced Composites Technology (ACT) program has adopted textile composites made by resin transfer molding-type processes and evaluated numerous applications in commercial transport airplanes [16]. Methods have also been developed to predict resin infiltration and flow in textile preforms and to predict and measure the mechanical properties of the textile composites. Andrew Beehag and Lin Ye [17] studied the parameters of manufacturing commingled CF/PEEK, and the tested flexural strength was 140 MPa. Honglin Luo et al. [18] studied the fabrication and characterization of three-dimensional carbon fiber-reinforced poly (ether ether ketone) (C3D/PEEK) composites for orthopedic applications. They found that the pre-heating of 3D fabrics before hot-pressing could eliminate pores in the composites prepared by 3D co-braiding and hot-pressing techniques. Also, the manufacturing process and the processing variables were studied and optimized. The optimized flexural strength was 500 MPa.

This study focuses on the preparation of a mixed flexible pre-prepreg to solve the manufacturing problems of complex components, reduce the buckling of CFs in molding, and ensure the uniformity of composite materials. In the present work, four significant parameters—melting temperature, molding pressure, crystallization temperature and resin mass contents—were investigated for the molding of CF/PEEK plain weave fabrics. It was the first time that CF/PEEK plain weave fabric was used to make composites and study the molding parameters. DSC, TGA and contact angle tests were used to test the melting range, thermal stability and the contact angle at high temperatures, respectively. An ultra-depth-of-field 3D microscope was adopted to research the effects of molding pressure in the melting stage. The mechanical properties of composites and crystallinity under different crystallization temperatures were studied. Three different contents of CF/PEEK plain weave fabrics were fabricated and used to discuss the effects of resin mass ratio on mechanical properties.

## 2. Materials and Methods

### 2.1. Materials

The commercially available PAN-based carbon fiber tows used in this study were T300B-3000-50B, with a diameter of 7.5 μm, purchased from Toray Industries Inc. (Tokyo, Japan). Commercially available fibrous PEEK resin (180D/36F), with an average diameter of 27 μm, was purchased from Changzhou CO-Win Novel Materials Co, Ltd. (Changzhou, China). These fibers were all washed with ethyl alcohol and acetone and then dried in a vacuum oven at 80 °C for 1 h to remove dust, grease and chemical residues before use.

**Figure 1:**
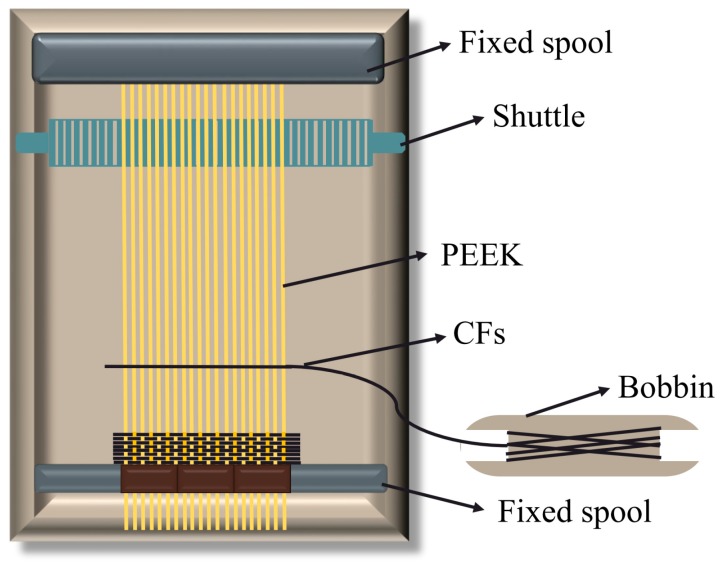
The schematic diagram for the fabrication of CF/PEEK plain weave fabric.

A semi-automatic proofing machine, Model SGA598, purchased from Jiangyin Tong Yuan Textile Machinery Co., Ltd. (Jiangyin, China) was used to fabricate the CF/PEEK plain weave fabric of three contents. As shown in Figure 1, PEEK fibers were used as warp threads and CFs as weft threads to avoid the break of monofilament caused by the friction of shuttle during the weave process. The warp densities were fixed at 50/10 cm for the three contents of CF/PEEK plain weave fabric: 6-strand, 12-strand and 18-strand PEEK fibers.

### 2.2. Hot Compaction

**Figure 2:**
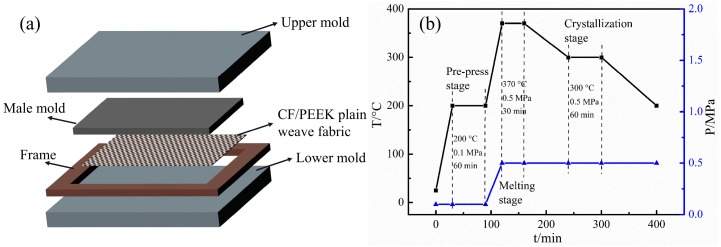
The mold diagram and molding procedure for the CF/PEEK plain weave fabrics: (**a**) The mold diagram; (**b**) the molding procedure.

The molds used in these experiments are shown in Figure 2a. CF/PEEK plain weave fabrics were placed layer by layer. For the molding of the flexible mixed premix, three stages of the heating process were adopted [19,20,21]. In the first stage, the pre-prepreg was preliminarily compacted by preloading to form the necessary shape of each layer. Resins were heated for softening and melting as a preparation for hot-pressing, and this process was sustained for a period of time to ensure that the layers were in close contact with each other and the layers were fully stretched [22]. This is the softening stage, and the temperature was usually set 50 °C higher than T_g_. The second stage was the melting process, which was maintained for enough time under an appropriate pressure to ensure that the resin was fully melted and impregnated with CF bundles. The last stage was crystallization, which caused the interface of the CF/PEEK to become tightly connected.

In accordance with the reported studies ([8,23]), we adopted the molding procedure shown in Figure 2b. For the study of pressure, the molding temperature and time were set according to the process system in Figure 1, while the pressure was set at 0.3, 0.5, 0.7 and 1.0 MPa. When discussing the effect of crystallization temperature, the temperature of the third stage needs to be adjusted to 290, 300, 310 and 320 °C with a fixed pressure of 0.5 MPa at the melting process. To study the effects of the PEEK contents, the manufacturing process was designed as shown in Figure 2b. Twenty-ply laminates of unidirectional and [0/90]_5s_ plates were molded to study the effects of the different process parameters. The unidirectional plates fabricated from 12-strand CF/PEEK plain weave fabrics were used for the study of crystallization temperature, and in the other parts, laminates of [0/90]_5s_ fabricated from 12-strand CF/PEEK plain weave fabrics were used.

### 2.3. Characterization

#### 2.3.1. Surface Morphology

An MSD-VHX1000 ultra-depth-of-field 3D microscope was used to characterize the two-dimensional topographic map of the plain weave fabric and its composite. Scanning electron microscope (SEM) (Merlin Compact, ZEISS, Dresden, German) measurements were utilized to observe the surface topography and provide a general analysis for the material after mechanical tests.

#### 2.3.2. Contact Angle Measurement

The static contact angles at different temperatures (360, 365, 370, 375 and 380 °C) were tested by a high-temperature wetting angle measuring instrument VAF-30 assembled by Harbin Institute of Technology. The tested PEEK cylinders of 3 × 3 mm were fabricated from PEEK fibers. The contact angle tested at each temperature was obtained by holding the cylinder stable for 30 min.

#### 2.3.3. Thermal Properties and Crystallinity

Differential scanning calorimetry (DSC) measurement was carried out on an STA449F3 synchronous thermal analyzer (NETZSCH, Selb, Germany) to study the melting range and crystallinity. The fibrous PEEK and CF/PEEK composites, cut into a small size with a weight of ∼10 mg, were thermally cycled between 25 °C and 400 °C twice at a constant heating/cooling rate of 10 °C min^−1^ in a nitrogen atmosphere [24]. The crystallinity *X_c_* was calculated by the following equation:

(1) $$X_{c}=\frac{\Delta H_{m}}{\Delta H_{m}^{0}\alpha }\times 100\%$$

where $\Delta H_{m}^{0}$ is the melting enthaply of 100% crystalline PEEK, $\Delta H_{m}^{0}$ = 130 J/g, and $\Delta H_{m}$ is the melting enthalpy of PEEK fibers tested by DSC. *α* is the mass fractions of PEEK.

The thermogravimetric analysis (TGA) tests for PEEK fibers with an approximate 10–15 mg were conducted in a nitrogen and air atmosphere from 25 °C to 800 °C at a heating rate of 10 °C min^−1^ to study the thermal stability of PEEK fibers [25].

#### 2.3.4. Resin Mass Contents

In this experiment, the content of resin was tested by the burning method. The basic principle to calculate the content of resin was based on the weight changes before and after burning, which was based on the characteristic that PEEK can be completely burned at high temperature, and the fibers were retained. After burning at 600 °C for 6 h in N_2_, PEEK was decomposed and the quality of the remaining CFs was obtained. The resin mass fraction was calculated by Equation (2):

(2) $$M=\frac{W-W_{0}}{W}\times 100\%$$

where *W* is the mass of the sample and *W*_0_ is the mass of the remaining CFs.

### 2.4. Mechanical Properties

#### 2.4.1. Tensile Tests

Tensile tests were performed on an Instron 5569 Electronic universal testing machine (Norwood, MA, USA), with hydraulic clamps and a 50 kN load cell at a speed of 2 mm/min. The load cell was electronically scaled to 0.1 kN to improve its accuracy. The tensile samples measured 250 by 25 mm and were tested at a gauge length of 150 mm, according to ASTM D3039. At least 5 samples were tested for each processing condition [26,27]. Sand paper was used in all cases to avoid slipping in the clamps. The strength was determined as the maximum stress in the tensile diagram and the corresponding strain was defined as the failure strain.

The tensile strength $\sigma_{T}$ and modulus $E_{T}$ are calculated by the following equations:

(3) $$\sigma_{T}=\frac{P_{max}}{bh}$$

where

*P_max_* is the maximum force carried by test coupon before failure (N),
*b* is the width of the beam (mm) and
*h* is the thickness of the beam (mm)

(4) $$E_{T}=\frac{\Delta \sigma_{T}}{\Delta \varepsilon_{T}\times 1000}$$

where

$E_{T}$ is the tensile modulus (GPa),
$\Delta \sigma_{T}$ is the tensile strength between two selected points (MPa),
$\Delta \varepsilon_{T}$ is the strain between the two selected points.

#### 2.4.2. Bending Tests

For residual bending strength estimation, 3-point bending was carried out using a 3-point fixture attached to a universal testing machine (Instron 5569) and the entire procedure was subject to the ASTM D7264 method at a speed of 1 mm/min. The size of the specimen was 75 × 12.5 × 2.2 mm and the span-to-depth ratio was controlled at 20:1. Five samples were tested to obtain the average values of bending strength and modulus. The values of stress were linearly proportional to the deflection of CF/PEEK specimens, and the bending stress was measured by assuming that the specimens were homogeneous isotropic materials [25].

The flexural strength and flexural chord modules of elasticity are calculated by the following equation:

(5) $$\sigma_{f}=\frac{3PL}{2bh^{2}}$$

where

$\sigma_{f}$ is the flexural strength (MPa),
*P* is the applied force (N),
*L* is the supported span (mm),
*b* is the width of the beam (mm) and
*h* is the thickness of the beam (mm).

(6) $$E_{f}^{chord}=\frac{\Delta \sigma_{f}}{\Delta \varepsilon \times 1000}$$

where $E_{f}^{chord}$ is the flexural chord modules of elasticity (GPa), $\Delta \sigma_{f}$ is the difference in flexural stress between the two selected strain points (MPa), and Δε is the difference between the two selected train points (nominally 0.002).

## 3. Results and Discussion

### 3.1. Determination of Molding Temperature

**Figure 3:**
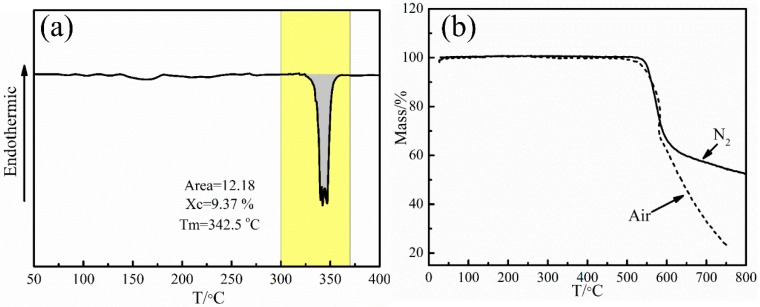
The DSC and TGA curves of PEEK fibers: (**a**) DSC curve of PEEK fibers in N_2_ and (**b**) TGA curves of PEEK fibers in air and N_2_.

The melting process is performed to make the PEEK fiber resin matrix fully infiltrate the CFs and squeeze bubbles under a certain pressure. Therefore, the molding temperature should be higher than the melting point and lower than the oxidation temperature, so that the resin will have better fluidity and infiltrate the carbon fibers well ([22,28]). For the structures of semicrystalline PEEK fibers, the melting range should be considered when selecting the molding temperature. DSC tests were applied to study the melting process of PEEK fibers, and the result is shown in Figure 3a. It can be seen that the melting range is from 320 to 360 °C and the temperature of the melting peak is at 342.5 °C. The calculated crystallinity X_c_ is 9.37%. Here, there are several melting peaks in the melting range for the semi-crystalline property of PEEK fibers. The melting points in different crystallinity regions are different [24].

In our study, the molding process was performed in air, and so the thermal stability of PEEK fibers in air should be studied. The PEEK fibers were tested by TGA in N_2_ and air atmospheres, and the experimental results are shown in Figure 3b. It is obvious that PEEK fibers began to lose mass rapidly at around 550 °C, which was caused by the production of low-boiling point benzene and phenol generated from the thermal degradation of the main chain of PEEK [25]. After 600 °C, the mass loss of PEEK fibers in the N_2_ atmosphere slowed down while the mass of PEEK fibers in the air atmosphere continued to decrease rapidly, due to the oxidation of PEEK and the gas generated by the pyrolysis of residual carbonized products in air. The quality of PEEK fibers did not change significantly before 520 °C, whether in N_2_ or in air, indicating that PEEK fibers were neither oxidized nor decomposed before 520 °C. Combined with the melting range of PEEK fibers, the melting temperature could be between 360 and 500 °C.

**Figure 4:**
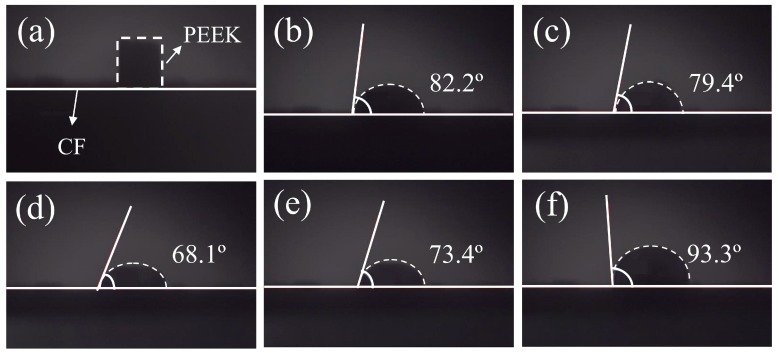
Static contact angles of PEEK on CFs under different temperatures: (**a**) 25 °C; (**b**) 360 °C; (**c**) 365 °C; (**d**) 370 °C; (**e**) 375 °C; (**f**) 380 °C.

The wettability between PEEK fibers and CFs can be characterized by the high-temperature contact angle. When the contact angle is less than 90°, the wettability between two substances is good; the smaller the contact angle is, the better the wettability is, and so when the contact angle is greater than 90°, the wettability between the two materials is poor. A cylindrical PEEK resin was placed on the surface of the CF cloth as shown in Figure 4a. The contact angles between the PEEK and CF cloth at different temperatures are shown in Figure 4b–f. It can be seen that after the melting of PEEK, the contact angles of PEEK and CF decrease as the temperature increases and reduce to a minimum of 68.1° at 370 °C, indicating the best wettability at this condition. However, when the temperature rises continuously, the contact angle increases, indicating the gradual deterioration of the wettability, and at 380 °C, the contact angle is 93.3°, showing non-wettability at this condition. The contact angles are closely connected with the surface energy of melted PEEK and CFs, and the surface energy of PEEK differs with the changes of temperature [29], because CFs cannot change their shape when the surface energy of melted PEEK changes. Liquids can shrink or spread during the process to maintain the balance among gas, liquids (melted PEEK) and solids (CFs). According to the minimum energy principle, if the surface energy of CF is larger than the surface energy of melted PEEK at a certain temperature, CFs will adsorb melted PEEK, so that molten PEEK will spread to reduce the contact area with gas and the solid surface energy, CFs will be wetted, and the contact angle will be small. In contrast, if the surface energy of CFs is lower than the surface energy of the melted PEEK, the solid surface adsorption will be weakened, and molten PEEK will shrink to reduce the surface energy of the system, such that the solid surface will not be wetted and the contact angle will be high [30]. By considering the contact angles between PEEK and CFs, the melting temperature was determined at 370 °C.

### 3.2. Effects of Molding Pressure

In the molding process of composites, pressure plays an important role in the properties of products. The thermoplastic matrix is a viscous fluid in the melting state, which is surrounded by a large number of bubbles. These bubbles must be discharged by applying pressure [17,31]. If the molding pressure is too low, a large number of defects will appear in the composite materials, as bubbles cannot be completely discharged from composites. If the pressure is too high, this will probably lead to resin outflow, loss of resin content, and possibly the extrusion deformation of carbon fiber bundles. As the new preform forming method adopted in this paper, PEEK and CFs were woven together. The carbon fibers were not restrained in the transverse direction, and during the molding process, CFs may be dispersed with the flow of PEEK resin under high pressure.

**Figure 5:**
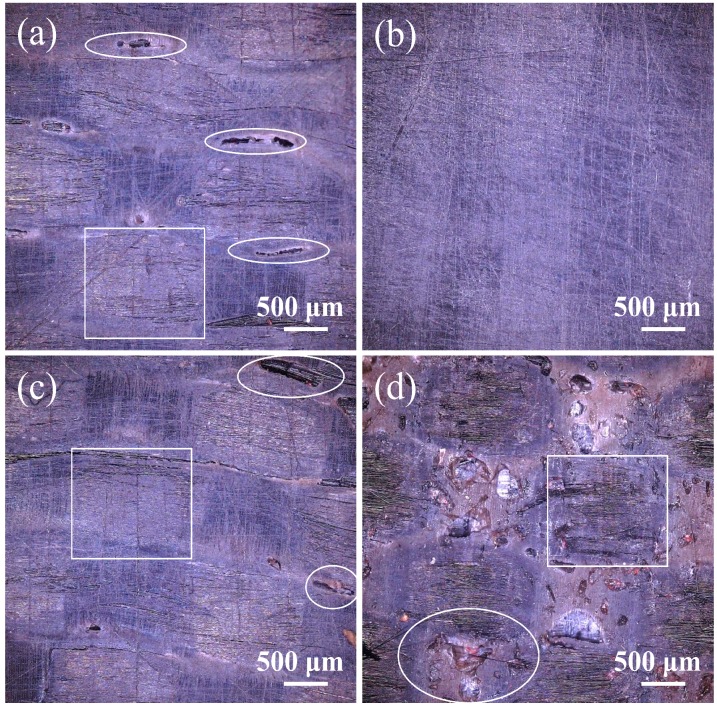
Surface morphology of CF/PEEK directional plates under different molding pressures: (**a**) 0.3 MPa; (**b**) 0.5 MPa; (**c**) 0.7 MPa; (**d**) 1.0 MPa.

After determining the molding temperature, directional plates with 20 layers of CF/PEEK plain weave fabric were molded under different pressures according to the process system described in Section 2.2. The surface morphologies of unidirectional plates under different pressures were observed by ultra-depth-of-field microscopy, and the results are shown in Figure 5a–d. When the pressure is 0.3 MPa, there are obvious connected pits on the surface of the unidirectional plate resulting from the aggregated bubbles in the resin. The bubbles are difficult to squeeze from the composite when the pressure is relatively low. It can be observed that a laminate with uniform surface was obtained at the pressure of 0.5 MPa, as shown in Figure 5b, which meant that this was an appropriate pressure to squeeze bubbles and form a uniform and stable composite. When the pressure increased to 0.7 MPa, there were some small defects resulting from the aggregated bubbles that failed to discharge with the increased flow speed of melted resin, and it can be seen that the shape of the CF tows was lightly deformed under this pressure. As the pressure increased, the flow rate rose, resulting in the defects becoming more obvious; the dispersion of CF tows can also be observed in Figure 5d. Thus, the molding pressure was 0.5 MPa.

### 3.3. Selection of Crystallization Temperature

The crystallization properties of polymers have significant effects on the properties of composites. Therefore, by adjusting the crystallization behavior of polymers, composites with excellent properties can be obtained ([32,33]). In this part, CF/PEEK composites were isothermally crystallized at 290, 300, 310 and 320 °C, and the mechanical properties and crystallinity of the composites were tested. The longitudinal tensile modulus *E*_1_, transverse tensile modulus *E*_2_, longitudinal tensile strength *X_t_* and transverse tensile strength *Y_t_* were tested, and the results are shown in Table 1. It can be seen that the longitudinal and transverse tensile moduli of all samples did not change significantly. The sample crystallized at 300 °C showed the highest longitudinal and transverse tensile strength, and the longitudinal tensile fractures are shown in Figure 5.

**Figure 6:**
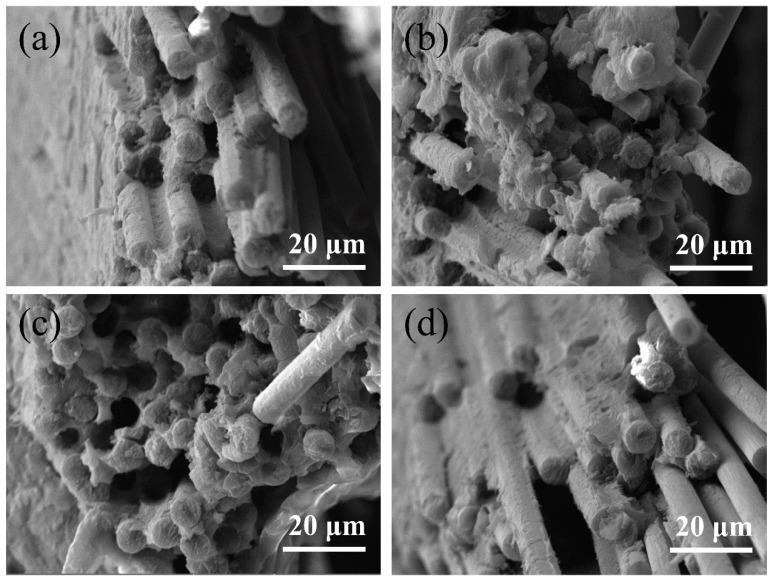
Longitudinal tensile fracture of CF/PEEK unidirectional plates at different isothermal temperatures: (**a**) 290 °C; (**b**) 300 °C; (**c**) 310 °C; (**d**) 320 °C.

When a unidirectional plate was subjected to longitudinal tensile stress, due to the larger elastic modulus of the CFs, the tensile stress of the carbon fibers was much greater than that of PEEK when both reached a certain strain at the same time. The stress on CFs was transferred to PEEK through the interface. If the interfacial shear strength between CFs and the PEEK resin is relatively small, the interface damage occurs first. The smaller the interfacial shear strength between the CFs and resin, the more serious the interfacial damage was, and the longer the fiber was pulled out. Finally, the fracture of CFs occurs due to the stress concentration and the CFs were pulled out, as shown in Figure 6c,d. On the other hand, if the average interfacial strength between the CFs and resins is high, the remaining interfacial strength after defective interfacial failure still has enough strength to allow the breakage of CFs to occur before a large area of interfacial failure [26,27]. Finally, a small number of CFs were pulled out from the matrix, as can be seen from the fracture area shown in Figure 6a,b.

**Figure 7:**
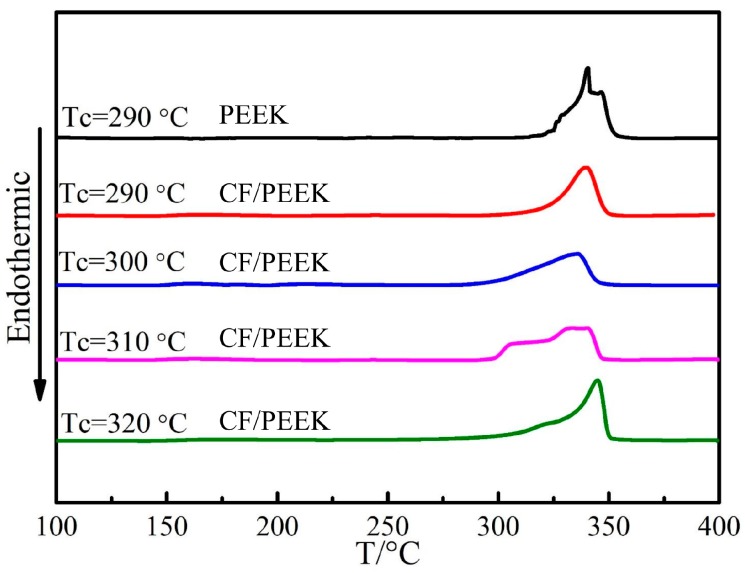
DSC curves of CF/PEEK fabricated at different crystallization temperatures.

The DSC curves are shown in Figure 7. In addition, in some curves, the phenomenon of double melting peaks is observed. The peak shape is a reflection for the crystallization and crystal size. As reported [33], the spherulite size of pure PEEK resin depends primarily on the crystallization temperature. Meanwhile, for CF/PEEK composite, the factors that determine the crystal size are temperature and the distance between two adjacent CFs, and the latter is a key factor. For pure PEEK crystallized at 290 °C, crystal defects or separate populations of crystals formed by isothermal and non-isothermal crystallization was the reason of double melting peaks reflected in DSC curves. At the same temperature, the addition of CFs decreased the energy needed for the formation of nucleation and provided a large amount of nucleation for PEEK. Therefore, the crystal defect or separate population of crystals was not obvious as before, which was reflected from the melting zone. In the crystallization process at 310 °C, the distance between two adjacent CFs controlled the growth and size of crystalline. The size of crystals developed from CFs differed significantly form that of bulk PEEK due to the high crystal growth rate under this temperature. At 320 °C, the undercooling and nucleation driving force was reduced when the crystallization temperature is high, it was difficult to nucleation in bulk PEEK, so the melting bimodal phenomenon was obscure.

Theoretically, these separate crystals should be considered in detail when calculating the total degree of crystallinity X_c_, such as performing a deconvolution of the peaks and considering two different reference enthalpies of fusion. Meanwhile, as reported [19], WAXS experiments performed in a previous study on PEEK samples demonstrated that both crystal populations correspond to the same crystalline structure. As a consequence, an identical enthalpy of fusion for both phases can be assumed, which agrees with the methodology used by many authors. The analysis results obtained from DSC tests are illustrated in Table 2. It can be concluded that the addition of CFs was propitious to increasing the matrix crystallization, and the higher the crystallization temperature, the higher the crystallinity. Although the undercooling and nucleation driving force are high when the crystallization temperature is low, the nucleation growth speed is slow due to the low temperature [20]. By considering the actual production efficiency, the isothermal crystallization time was set for 1 h. After isothermal crystallization, the non-isothermal crystallization stage was conducted by natural cooling, and so the higher the isothermal crystallization temperature, the higher the crystallization rate in both stages. Combining the tensile results with crystallization, the temperature was set at 300 °C.

### 3.4. Effect of Resin Content

**Figure 8:**
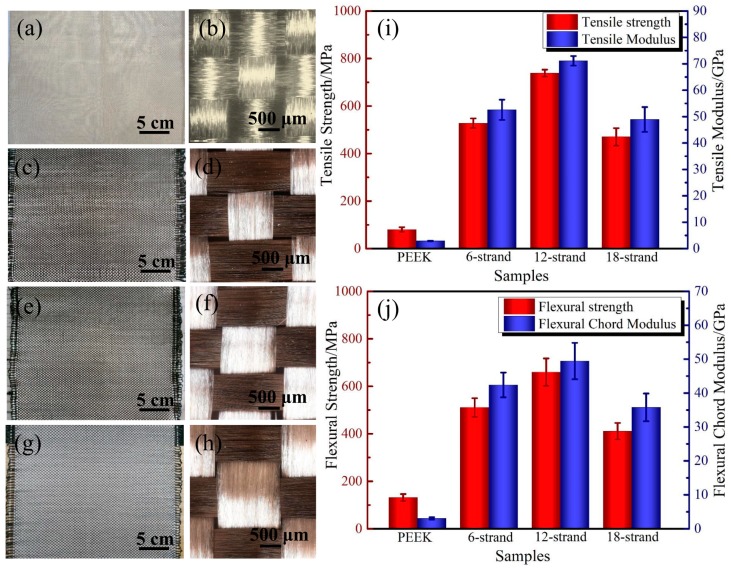
Surface morphology of PEEK and CF/PEEK plain weave fabrics of different contents and the mechanical properties of composites: (**a**) PEEK plain weave fabrics; (**b**) PEEK plain weave fabrics; (**c**) plain weave fabrics with 6-strand PEEK fibers; (**d**) plain weave fabrics with 6-strand PEEK fibers; (**e**) plain weave fabrics with 12-strand PEEK fibers; (**f**) plain weave fabrics with 12-strand PEEK fibers; (**g**) plain weave fabrics with 18-strand PEEK fibers; (**h**) plain weave fabrics with 18-strand PEEK fibers; (**i**) the tensile strength and modulus and (**j**) the flexural strength and modulus.

The morphologies of the PEEK fabrics and CF/PEEK plain weave fabrics with different contents are shown in Figure 8a–h. The mass ratios of the PEEK resin in CF/PEEK composites were calculated according to the method described in Section 2.3.4, and the mass ratios were 50.45%, 59.07% and 65.32% for 6-strand, 12-strand and 18-strand, respectively. Figure 8i shows the tensile properties, including the strength and modulus of pure PEEK and CF/PEEK composites with different PEEK mass ratios. It is obvious that the strength and modulus are relatively low when compared with CF/PEEK composites. The tensile strength and modulus are 79.37 ± 12.59 MPa and 2.79 ± 0.44 GPa respectively. It can be seen that the tensile strength and tensile modulus of samples with 12 strands are higher than others, as a reasonable ratio of CF and PEEK can reduce the defects in the forming process and transfer stress in an efficient way. The tested tensile strength and modulus of CF/PEEK composites with a 59.07% resin mass ratio were 738.36 ± 14.49 MPa and 71.12 ± 1.79 GPa, respectively, which was a significant improvement with respect to pure PEEK. Figure 8j illustrates the flexural properties of PEEK and CF/PEEK composites. For pure PEEK molded from PEEK fabrics, the flexural strength and modulus are 131.23 ± 14.78 MPa and 3.07 ± 0.32 GPa. The best flexural properties were obtained in CF/PEEK composites with a 59.07% resin mass ratio. The flexural strength and chord modulus were 136.25 ± 23.8 and 3.07 ± 0.56 GPa. Table 3 listed the mechanical properties of CF/PEEK fabricated from 3D braided hybrid CF/PEEK fabric, commingled CF/PEEK unifabric, PEEK powder and films. For 3D braided hybrid CF/PEEK fabric, the damage of CFs caused by the weave process, crimp and the stack of CFs in the molding process are reasons for the relatively low flexural properties. Actually, the commingled CF/PEEK unifabric and cowoven hybrid fabrics are really excellent methods for the tight combination of CFs and PEEK fibers in one tow. However, these fabrics are difficult to prepare in experiments. For PEEK powders and films, the low mobility and high melt viscosity of PEEK make it difficult to fully infiltrate CFs, resulting in the relatively low mechanical properties of its composites.

**Figure 9:**
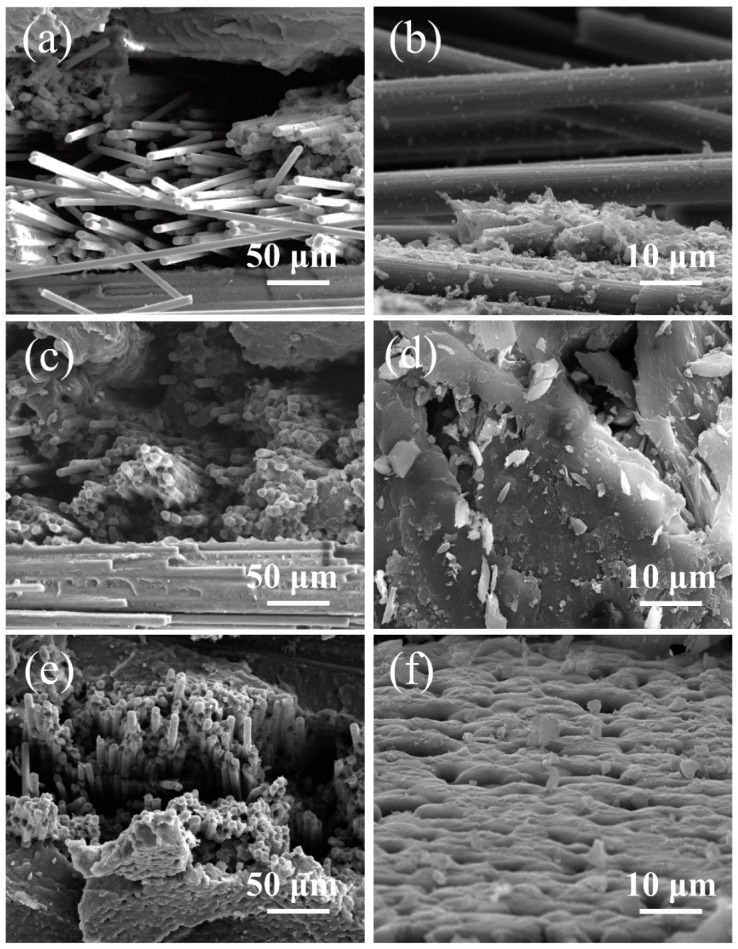
The morphology of CF/PEEK composites with different PEEK mass ratio after the tensile test: (**a**) Fracture morphology of composites with 6-strand PEEK fibers; (**b**) The morphology of CFs in composites with 6-strand PEEK fibers; (**c**) Fracture morphology of composites with 12-strand PEEK fibers; (**d**) The morphology of PEEK resin in composites with 12-strand PEEK fibers; (**e**) Fracture morphology of composites with 18-strand PEEK fibers; (**f**) The morphology of PEEK resin in composites with 18-strand PEEK fibers.

Figure 9 illustrates the morphology of samples with different resin contents after tensile fracture tests. For the samples after fracture test that were not of uniform height, it was difficult to observe the target part under different magnifications. Here, Figure 9a,c,e were in the same magnification to show the morphology of samples with different resin contents after tensile fracture test. Figure 9b,d,f were in the same magnification to observe the different parts of samples with different resin contents after tensile fracture test. If the resin content is relatively low, resin is not able to fully infiltrate CFs, leading to an interface with insufficient strength to hinder the transfer of stress, which can be found in the morphology of the tensile fracture shown in Figure 9a,b. On the contrary, composites with a high resin content will have relatively poor properties due to their low CF content and the defects caused by resin flow, as shown in Figure 9e,f. A large number of pores are found in Figure 9f, and these defects can result in the invalidation of stress transfer and a decrease in interfacial strength. Figure 9c,d shows the tensile fracture of samples with a 59.07% resin mass content, and we can see that the CFs were fully infiltrated by PEEK resin, with a uniform distribution of resin, resulting in high tensile properties [34].

## 4. Conclusions

In this work, four parameters were investigated for hot-compacted CF/PEEK plain weave fabric composites: melting temperature, molding pressure, crystallization temperature, and, finally, the mass content of resin. Traditionally, a higher compaction temperature leads to more matrix creation and better layer bonding, but here we considered the melting temperature from the wetting behavior of PEEK on CF mats. Melting temperature had a non-negligible effect on the static contact angle of PEEK on CF mats, ranging from 68.1° to 93.3°, and the smaller the contact angle, the better the wettability. A suitable molding pressure causes bubbles to be discharged from composites and helps to form a uniform surface. An ultra-depth-of-field 3D microscope was adopted to research the morphologies of composites molded under different pressures in the melting stage, and composites molded at 0.5 MPa produced a well-distributed surface with no defects. Samples crystalized at 300 °C showed excellent tensile properties and crystallinity. Resin mass content is also an important parameter in the fabrication of composites. Three contents of CF/PEEK plain weave fabrics were prepared, and the mass contents were tested by the burning method. Increased mass ratios of PEEK ranging from 50.45% to 59.07% allowed for much stronger interfacial strength; however, a higher resin content could lead to the dispersion of CFs, loss of resin and the formation of defects during processing. Finally, the optimal resin mass content was 59.07%, with a tensile strength of 738.36 ± 14.49 MPa and a flexural strength of 659.68 ± 57.53 MPa.

The experiments show that PEEK fibers and CF can be used to prepare blended flexible pre-prepreg, and this method fills in the blank of CF/PEEK composites made of plain weave fabric, expanding their application in the field of complex component preparation. This paper studied the optimized processing parameters to obtain better properties from CF/PEEK plain weave fabrics and broaden the future specific applications of CF/PEEK composites, demonstrating a new direction for its fabrication. Further investigation will include the wettability of CF and PEEK fibers and the modification of CF/PEEK plain weave fabric.

## Figures and Tables

**Table 1:** Longitudinal and transverse tensile properties of CF/PEEK unidirectional plates at different isothermal temperatures. (*X_t_* is the longitudinal tensile strength; *E*_1_ is the longitudinal tensile modulus; *Y_t_* is the transverse tensile strength; *E*_2_ is the transverse tensile modulus).

Isothermal Temperature (°C)	*X*_t_ (MPa)	*E*_1_ (GPa)	*Y*_t_ (MPa)	*E*_2_ (GPa)
290	1301.54 ± 36.09	128.99 ± 9.73	47.63 ± 8.29	8.78 ± 1.70
300	1395.67 ± 95.84	132.32 ± 9.41	51.25 ± 10.01	8.86 ± 2.14
310	1380.91 ± 109.85	133.33 ± 11.24	46.94 ± 6.01	8.91 ± 1.03
320	1374.30 ± 91.27	135.83 ± 18.57	43.28 ± 5.67	8.88 ± 1.82

**Table 2:** DSC results for different samples. (*T* is the tested crystallization temperature; Δ*H*_*m*_ is the enthalpy; *X*_*c*_ is the crystallinity).

*T* (°C)	Material	Resin Mass Content (%)	Δ*H*_*m*_ (J/g)	*X*_*c*_ (%)
290	PEEK	100	38.66	29.74
290	CF/PEEK	59.07	31.00	40.38
300	CF/PEEK	59.07	33.51	43.64
310	CF/PEEK	59.07	37.03	48.21
320	CF/PEEK	59.07	45.04	58.66

**Table 3:** Longitudinal and transverse tensile properties of CF/PEEK unidirectional plates at different isothermal temperatures. (σ**_*t*_** is the tensile strength; *E***_*t*_** is the tensile modulus; σ**_*f*_** is the tensile strength; *E***_*f*_** is the tensile modulus).

Samples	Method	σ_*t*_ (MPa)	*E*_*t*_ (GPa)	σ_*f*_ (MPa)	*E*_*f*_ (GPa)
CF/PEEK	3D braided hybrid CF/PEEK fabric [18]	-	-	456.2 ± 15.6	41.0 ± 3.5
CF/PEEK	Commingled CF/PEEK unifabric [17]	-	-	125.0 ± 25.0 (transverse)	10.0 ± 0.9 (transverse)
CF/PEEK	PEEK powder [5]	-	-	160	-
CF/PEEK	Cowoven hybrid fabrics [6]	-	-	700	-
CF/PEEK	PEEK films [9]	111 (transverse)	3.9 (transverse)	-	-
